# Bronchogenic cyst of the neck in an elder patient: A case report

**DOI:** 10.1016/j.ijscr.2019.10.013

**Published:** 2019-10-12

**Authors:** Inês Santos, João Barros, Teresa Lopes, Margarida Mesquita, Leonor Barroso, Isabel Amado

**Affiliations:** Maxillofacial Surgery Department, Hospital and University Centre of Coimbra, Praceta Prof. Mota Pinto, 3000-075, Coimbra, Portugal

**Keywords:** Bronchogenic cyst, Duplication cyst, Neck, Case report

## Abstract

**Introduction:**

Bronchogenic cysts are rare malformations, mostly diagnosed in children. We report the rare case of a neck bronchogenic cyst diagnosed in an elderly patient.

**Presentation of case:**

The patient complained of a long-standing submental mass. The diagnostic work-up resulted in a thyroglossal duct cyst diagnosis for which the patient underwent a Sistrunk procedure. However, the histological analysis of the lesion ultimately revealed a bronchogenic cyst.

**Discussion:**

Neck bronchogenic cysts are rare and, in adults, normally asymptomatic. Imaging exams can suggest the diagnosis but they are most important for surgical planning. Surgery is the elected treatment for bronchogenic cysts and the histopathologic exam of the specimen provides definitive diagnosis.

**Conclusion:**

This case demonstrates than even though they are a rare diagnosis, bronchogenic cysts should be considered in the diagnostic work-up of neck cysts, even in elderly patients.

## Highlights

• Bronchogenic cysts are rare foregut duplications cysts.
• Mostly diagnosed in children, they may be found in elder patients.
• Neck bronchogenic cysts, albeit unusual, should be considered in the differential diagnosis of cystic masses of the neck.
• Surgery is the treatment of choice for bronchogenic cysts.

## 1 Introduction

Bronchogenic cysts are exceptional congenital malformations derived from the primitive ventral foregut [[1], [2], [3], [4]]. They are usually found in an intrathoracic location, either the mediastinum or lung parenchyma. However, there have been reports on other infrequent locations, such as the neck [1,[5], [6], [7]]. Bronchogenic cysts are more commonly diagnosed in the paediatric population and seldom in adults [1,4,5,8].

Differential diagnoses include a plethora of congenital malformations and tumours. Even though imaging exams can be helpful in surgical planning and may suggest the hypothesis of a bronchogenic cyst, definitive diagnosis is made only by histopathological exam [3,6,9,10]. We report the case of a rare neck bronchogenic cyst, treated in the University Hospital of Coimbra, Portugal. This lesion had remained undiagnosed for more than 8 decades, and the histopathological analysis was crucial for the diagnosis. This work has been reported in line with the SCARE criteria [11].

## 2 Presentation of case

**Fig. 1:**
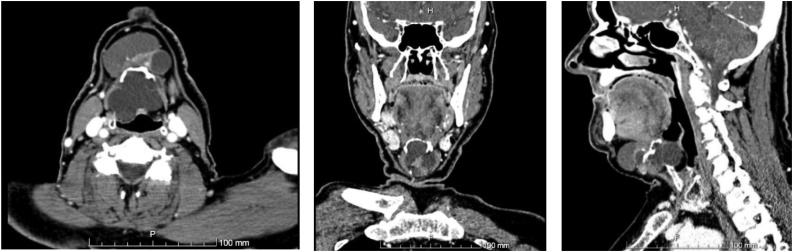
CT scan.

**Fig. 2:**
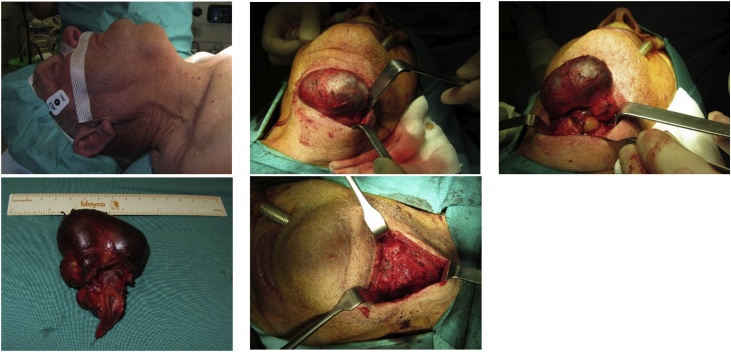
Surgery: a. submental mass; b. and c. dissection of the cyst; d. surgical specimen; e. cyst’s removal bed.

**Fig. 3:**
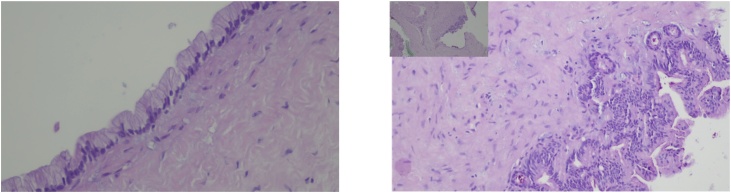
Histopathology - bronchogenic cyst lined by a ciliated columnar epithelium with seromucinous glands and a fibrous wall. a. ciliated columnar epithelium (H&E, 400×); b. seromucinous glands (H&E, 40×/200×).

**Fig. 4:**
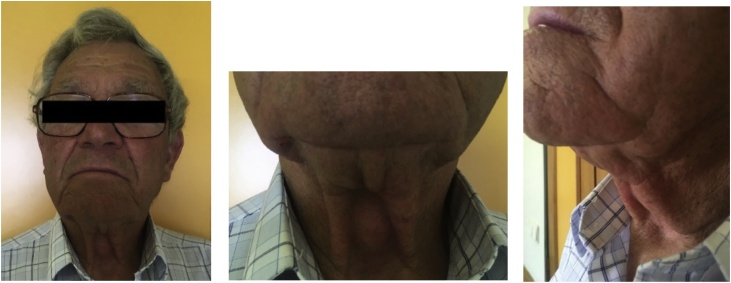
5 months follow-up.

An 84-year-old male was referred by his family doctor to our department for a long-standing submental mass. It caused discomfort and the patient reported several previous infections. Past medical history included a pulmonary embolism, peripheral venous insufficiency, heart failure and arterial hypertension. The diagnostic work-up included a CT scan on the neck (Fig. 1), revealing a median, infrahyoid cystic mass, which was multilocular and almost 7 cm in diameter. The lesion caused bony erosion to the hyoid and a deformity to the thyroid cartilage. The main diagnostic hypothesis was a thyroglossal duct cyst, for which the patient underwent surgery, ten months after presenting to our department. Under general anaesthesia, a Sistrunk procedure was performed by a maxillofacial surgery trainee, supervised by a maxillofacial surgeon (Fig. 2). The histopathological exam showed a multilocular cyst lined by a ciliated columnar epithelium with seromucinous glands and a fibrous wall (Fig. 3). Therefore, these findings established the diagnosis of a bronchogenic cyst. The patient had no complications and no local recurrence at 18 months of follow-up (Fig. 4).

## 3 Discussion

Bronchogenic cysts are rare malformations. During the fifth week of embryogenesis, the primitive foregut divides into a ventral trachea and dorsal oesophagus. The successive division of the ventral trachea forms the primitive lung tissue. However, if an aberrant budding from the bronchotracheal tree occurs, a bronchogenic cyst is formed [1,5,6,12,13].

They are usually a paediatric diagnosis, rarely occurring in adults. In fact, McAdams stated that presentation beyond 50 years of age is distinctly unusual [14]. They are more common in males, with a 4:1 ratio [4,15]. Most bronchogenic cyst are located in the thorax, but cervical cysts have been reported. The presence of these cysts in a cervical location can be explained by several theories [16]. Budding from an atypical upper location in the trachea could originate a cervical bronchogenic cyst [6,17]. Also, an intrathoracic cyst could undergo superior migration and become located in the neck [4]. These cysts are believed to result from either a distant migration of sequestered respiratory primordial cells during the organogenesis stage or to an superior movement of a preformed thoracic cyst caused by the formation of the sternum at a later stage [1,2,9,13,18,19]. Cervical bronchogenic cysts are, thus, usually located in the lower neck [9,10], with upper neck cysts being comparatively rare [20].

On January 2019, we searched PubMed for relevant publications on cervical bronchogenic cysts with abstracts in English. To be included, publications had to report cases of neck bronchogenic cysts in adults. Locations such as intramedullary, laryngeal or cutaneous were excluded from the review. Articles written in languages other than English, Spanish or French were also excluded. This led to the retrieval of 33 articles that included case reports of 55 neck bronchogenic cysts in adults. Unfortunately, one of these articles was not available, which left 53 cases of bronchogenic cysts. Of these, one case was furthermore excluded since it reported the case of an intrathoracic cyst with a neck location only with Valsalva manoeuvre (Table 1). In three cases, sex and age was not specified [3]. Of the remaining 49 patients, 18 were male and 31 were female. It seems that cervical bronchogenic cysts might be the exception to male predominance in this pathology. Regarding age of initial diagnosis, it was comprehended between 18 and 70 years old, with most cases (31/49) diagnosed before the age of 50. In what concerns location, most cases involved the lower neck (44/52), with only 8 cysts located in the upper neck. Of these, half were laterocervical and half were located in the midline. Interestingly, except for one case, whose age was not specified, all remaining three cases of upper midline bronchogenic cysts were diagnosed before the age of 30. This contrasts with our patient’s age. Lower neck cysts, on the other hand, were mostly located in the midline (38/44). This means that considering both lower and upper cysts, the majority are located in the midline (42/52). This preference of cervical bronchogenic cysts for the midline was already described by Bhattacharya and Crespo del Hierro, who stated that approximately 75% of cervical bronchogenic cysts were located in the midline [13,17].

**Table 1:** Neck bronchogenic cysts in adults reported in literature.

Reported cases of bronchogenic cysts in adults				
	Article	Age	Sex	Location
1	Dubois (1981) [25]	24	F	Supraclavicular region
2	McManus (1984) [22]	34	M	Between right carotid sheath and tracheo-oesophageal groove; beginning at cricoid cartilage and extending into mediastinum
3	Barsotti (1998) [26]	49	M	Posterior to the left lobe of thyroid gland
4	Rapado (1998) [27]	54	M	Supraclavicular triangle
5	Majilis (1999) [28]	44	M	Pretracheal anterior region
6	Shimizu (2000) [23]	25	F	Thyroid
7	Hadjihannas (2003) [6]	70	M	Suprasternal notch
8	Sanli (2004) [29]	48	F	Right paratracheal, beneath the thyroid lobe
9	Newkirk (2004) [8]	20	F	Thyroid
		22	M	Right paratracheal region
10	Al-kasspooles (2004) [1]	62	M	Supraclavicular region
11	Bocciolini (2006) [5]	57	M	Right paratracheal region
12	Ibañez Aguirre (2006) [30]	26	M	Thyroid
13	Shimazu (2006) [20]	22	F	Upper midline, in relation to hyoid
14	Eng (2006) [31]	41	F	Right level II
15	Markogiannakis (2008) [32]	52	M	Right superior parathyroid gland location
16	Moz (2009) [10]	39	M	Inferior to the right submandibular gland
17	Ergin (2009) [33]	28	M	Suprajugular notch
18	Hazenberg (2010) [16]	51	F	Dorsal to the right thyroid lobe
19	Calzada (2011) [2]	32	F	Thyroid
20	Niño-Hernandez (2011) [34]	29	F	Median neck mass in relation to the hyoid
21	Annamalai (2011) [12]	30	M	Thyroid
22	Crespo del Hierro (2013) [17]	67	F	Left level IIA
23	Yang (2013) [24]	67	F	Suprasternal region
24	Jun (2014) [35]	37–69	14 F	14 Paratracheal
			3 M	1 Thyroid cartilage
				1 Thyroid
				1 Right level III
25	Zaimi (2014) [36]	33	F	Inferior medial region
26	Jiang (2015) [3]	Non-specified	Non-specified	1 Upper midline
				1 Supraclavicular region
				1 Thyroid
27	Bhattacharya (2015) [13]	48	F	Supraclavicular region
28	Ramos (2015) [7]	45	F	Laterocervical, below the right lobe of the thyroid
29	Liu (2016) [37]	70	F	Below right thyroid gland
30	Farid (2017) [38]	24	M	Suprasternal region
31	Lee (2017) [39]	18–44	2 F	2 Infrahyoid
			1 M	1 Suprahyoid

These cysts are usually unilocular, fluid-filled and have no communication with the airway [1,4,5,13]. They normally persist unnoticed in adults. They become symptomatic, however, either due to mass effect or due to infection [2,3,6]. The compression of surrounding structures can result in dyspnea, cough and dysphagia [1,15,16]. Infection of deep cysts can originate an abscess, and superficial ones can result in fistulation [6,13,18,21,22]. The most serious complication, albeit extremely rare, is malignant transformation [21].

The differential diagnosis comprises other malformations such as branchial cleft cysts, thyroglossal duct cysts, cystic teratomas, cystic hygromas, lymphangiomas, epidermal and dermoid cysts, laryngoceles, ranulas, oesophageal duplication cysts and tracheal diverticula. Furthermore, it includes thymic and thyroid cysts, lipomas, parathyroid cysts, cystic neuromas, cystic papillary carcinoma of the thyroid gland and cystic degeneration of a lymph node [1,3,5,6,[8], [9], [10], [11], [12],^15^,^16^,^21^].

Aspiration cytology is an invaluable diagnostic tool in neck masses. Nevertheless, in bronchogenic cysts, aspiration cytology has a low sensitivity since only a few diagnostic cells may be found [13,24]. CT scan helps with the precise location of the cyst and its relation to adjacent neck structures, allowing for a more accurate planning of the surgical excision [1,17]. These lesions appear as round, well-circumscribed masses, with either water or soft tissue attenuation [5,14]. They can also include heterogeneous shadows due to calcium concentrations within the cyst [23]. However, it has been reported that MRI is the imaging modality of choice, because of its better soft tissue definition [13]. It also has the advantage of not needing intravenous contrast administration [10,18]. Bronchogenic cysts are shown as high-intensity lesions both in T1 and T2 weighted studies [17].

Even so, a definitive diagnosis can only be made upon histological analysis of the lesion [3,6,9,10]. As one would expect from its embryologic origin, a bronchogenic cyst is lined with ciliated, pseudostratified, columnar, respiratory-type epithelium, smooth muscle, hyaline cartilage and seromucinous glands [4,9,10,13,15,17]. However, there is not a consensus in the literature on the histological diagnostic criteria for a bronchogenic cyst. In fact, several authors have stated that the presence of every element is not necessary to classify the cyst as bronchogenic [1,8,16,17,20,23]. Maung has reported that cartilage is seldom present [9]. According to Hazenberg, frequently only respiratory epithelium is found in cervical bronchogenic cysts [16]. However, as has been described by Crespo del Hierro, although both thyroglossal duct cysts and branchial cleft cysts may have respiratory epithelium, they lack cartilage, smooth muscle and seromucinous glands [17]. Hence, it seems insufficient to rely solely on the existence of respiratory epithelium to make the diagnosis. Teissier and Ustundang, on the other hand, consider the presence of hyaline cartilage to be required to classify the lesion as a bronchogenic cyst [15,19].

Surgical excision is the elected treatment in adults, even in asymptomatic patients [1,3,4,6,9,10,13,17]. It allows for a definitive diagnosis, while avoiding symptoms or serious complications such as haemorrhage, infection or compressive symptoms. These develop in approximately 45% of asymptomatic patients [[1], [2], [3],^5^,^17^,^23^]. Furthermore, in the neck, resection procedures have a low morbidity rate [5,8]. Conservative treatment (watch-and-wait and percutaneous catheter drainage) should be reserved for high-risk patients [14,21]. Complete resection, through a transcervical approach, provides definitive treatment. In these cases, recurrence is unlikely [10,18]. However, when an incomplete resection is undertaken, a longer follow-up may be required [9].

Our case is, to the best of our knowledge, the eldest reported case in the literature. It is also one of the few upper neck location cysts described. Having a long-standing lesion, this patient had been symptomatic and had received several courses of antibiotic for recurrent infections. These previous infections were the reason we decided for surgical treatment, as the patient’s age and comorbidities would make him one of the few cases in which conservative treatment would be a valid treatment. Contrarily, if it were not for surgical excision, the correct diagnosis would not have been made, as the patient was clinically thought to have a thyroglossal duct cyst. The histological diagnosis was based on the presence of both respiratory epithelium and seromucinous glands.

## 4 Conclusion

Although bronchogenic cysts are rare, head and neck surgeons should bear in mind this hypothesis when dealing with a cystic neck mass, even in elderly patients.

Head and neck pathology comprises a multitude of diagnoses, many of which are uncommon, and in many instances, only the histopathological exam will settle the final diagnosis, underlining the value of a close collaboration with the Pathology department. Bronchogenic cysts are a perfect example of this since their differential diagnoses include several much more frequent disorders such as thyroglossal duct cysts.

## Sources of funding

None.

## Ethical approval

It is not a research study.

## Consent

Consent has been given.

## Registration of research studies

It was not a research study.

## Guarantor

Inês Santos; Isabel Amado.

## Provenance and peer review

Not commissioned, externally peer-reviewed.

## Declarations of Competing Interest

None.

## CRediT authorship contribution statement

**Inês Santos:** Data curation, Writing - original draft, Writing - review & editing. **João Barros:** Resources, Writing - original draft, Writing - review & editing. **Teresa Lopes:** Resources, Conceptualization, Methodology, Writing - review & editing. **Margarida Mesquita:** Conceptualization, Methodology, Writing - review & editing. **Leonor Barroso:** Resources, Formal analysis, Supervision, Writing - review & editing. **Isabel Amado:** Formal analysis, Writing - review & editing, Validation.
